# Special Issue “Structure, Activity, and Function of Protein Methyltransferases”

**DOI:** 10.3390/life12030405

**Published:** 2022-03-10

**Authors:** Arunkumar Dhayalan, Albert Jeltsch

**Affiliations:** 1 Department of Biotechnology, Pondicherry University, Puducherry 605014, India; 2 Institute of Biochemistry and Technical Biochemistry, University of Stuttgart, Allmandring 31, 70569 Stuttgart, Germany

Post-translational modifications (PTMs) largely expand the functional diversity of the proteome [1]. Protein methylation is an essential PTM, which regulates numerous cellular events by altering the functionality of proteins [2]. The methylation of histones regulates the chromatin structure and participates in the epigenetic regulation of gene expression in diverse biological processes, including development and differentiation [3]. Methylation also controls the activity of numerous non-histone proteins [2,4], where it often plays key roles in the regulation of their (i) stability, (ii) enzymatic activity, (iii) sub-cellular distribution, and (iv) interactions with other proteins. Aberrant protein methylation is implicated in various pathologies, including cancers [5,6].

**Figure 1:**
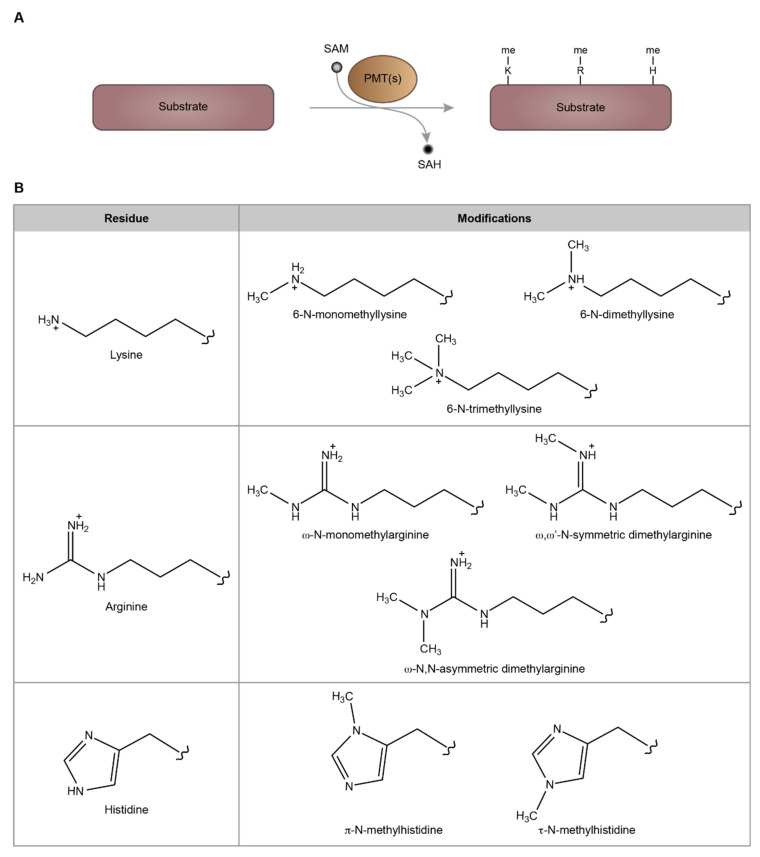
Methylation of proteins by PMTs. (**A**) Schema depicting the methylation of proteins by various PMTs at lysine (K), arginine (R), and histidine (H) residues. (**B**) The possible types of methylation modifications at lysine, arginine, and histidine residues. SAM, S-adenosyl-L-methionine; SAH, S-adenosyl-L-homocysteine.

Protein methylation mainly occurs at lysine and arginine residues and is catalyzed by protein lysine methyltransferases (PKMTs) and protein arginine methyltransferases (PRMTs), respectively. Protein methylation also occurs at the N-terminal α-amino group of proteins and other amino acids such as histidine and glutamine [4] (Figure 1). PKMTs methylate the lysine residues of proteins at three different levels and generate (i) monomethyllysine, (ii) dimethyllysine, and (iii) trimethyllysine (Figure 1). PKMT activity is exhibited by two protein domain families: (i) the SET domain-containing enzymes and (ii) the seven β strand domain-containing enzymes (7BS) [7,8]. The 7BS methyltransferase family is larger and contains several enzymes that methylate a wide range of substrates including arginine residues, DNA and RNA, in addition to the lysine residues [4,9]. It was predicted that the human genome encodes more than 100 PKMTs [10]. In contrast, PRMTs are only found in the 7BS family. The human genome contains at least nine different PRMTs, which are grouped into three types based on the nature of the methylarginine produced upon their enzymatic activity on arginine residues. Type I, II, and III PRMT enzymes catalyze the formation of asymmetric dimethylarginine, symmetric dimethylarginine, and monomethylarginine, respectively [11,12] (Figure 1).

A PubMed search for the terms “methylation” AND “lysine”, “methylation” AND “arginine”, or “methylation” AND “histidine” resulted in 622, 202 and 20 publications, respectively, for the year 2021 alone, suggesting that protein methylation research is a very exciting and active area of research. The goal of this thematic Special Issue is to collect and compile focused reviews about individual PMTs written by specialists in the field, which, according to our literature research, is currently not available for the majority of PMT enzymes. All review articles in this Special Issue cover central topics such as structure, biochemistry, cellular functions, and association with diseases, if any, and provide future perspectives. This Special Issue addresses an urgent demand in the field; currently, reviews on PMTs often analyze several unrelated enzymes, so the details and peculiarities of each one are not explored. The collection in this Special Issue, hopefully, will become a useful resource for researchers in the entire protein methylation and protein methyltransferase field.

Altogether, this Special Issue contains nine articles on individual PKMTs, six articles on individual PRMTs, and one article on the SETD3 histidine methyltransferase. In the context of PKMTs, Weirich et al. [13] reviewed the PKMTs SUV39H1 and SUV39H2 thoroughly, with a special focus on their substrate specificity profiles and non-histone protein substrates. Tauchmann and Schwaller [14] provided a review on the NSD1 PKMT, with detailed coverage on the role of NSD1 in developmental disorders and cancers. Rathert [15] presented the current literature status of the NSD3 PKMT, including structural aspects and the role of NSD3 in cancers. Klonou et al. [16] detailed the subunit composition of the MLL2 protein complex and discussed the role of the MLL2 complex in transcription and cellular functions. Poulard et al. [17] provided an elaborate overview of the G9a PKMT, covering various aspects, including structural features, non-histone substrates of G9a, the role of G9a on chromatin regulation, and its function in development, DNA repair, and diverse types of cancers. Markouli et al. [18] reviewed the structural, biochemical, and functional aspects of SETDB1 PKMT and discussed the role of SETDB1 in the physiological processes such as cell division, the formation of Promyelocytic leukemia nuclear bodies (PML-NBs), and the development of the nervous system and pathological conditions such as various types of cancers, neuropsychiatric diseases, genetic diseases, cardiovascular, and gastrointestinal diseases. Tellier [19] provided a review on various aspects of the SETMAR PKMT and discussed the role of SETMAR in non-homologous end joining (NHEJ) DNA repair pathway, restarting the collapsed replication fork, and chromosome decatenation in a detailed manner. Daks et al. [20] described the structure, substrate specificity, and cellular functions of SET7/9 PKMT, with particular focus on non-histone substrates and its role in cell proliferation and stress response. Finally, Jakobsson [21] covered the structural and biochemical features of the dual lysine methyltransferase METTL13, which is capable of catalyzing both N-terminal and lysine methylation, and highlighted its role in the regulation of global translation dynamics.

In addition to the above-mentioned PKMTs, this Special Issue also contains review articles about different PRMTs. Thiebaut et al. [22] reviewed different aspects of the major type I protein arginine methyltransferase PRMT1 and discussed the non-histone substrates of PRMT1, the role of PRMT1 in various cell signaling pathways, and DNA repair extensively. Cura and Cavarelli [23] provided a review on PRMT2, focusing on its structural features and its role in splicing. Motolani et al. [24] reviewed the functionally versatile major type II protein arginine methyltransferase PRMT5, focusing on its role in various human diseases such as various types of cancers, diabetes, cardiovascular, and neurodegenerative diseases. Gupta et al. [25] presented the current literature status of PRMT6 and covered topics such as structural features, kinetic mechanism, epigenetic functions, and non-histone substrates of PRMT6. Halebelian and Barsyte-Lovejoy [26] provided a comprehensive overview on various aspects of type III protein arginine methyltransferase PRMT7 and elaborated its role on gene expression, genome maintenance, pluripotency, differentiation, senescence, and stress response. Dong et al. [27] wrote a detailed review on the neuronal functions of the PRMT8, which is expressed exclusively in the brain, and discussed its potential role in neurological diseases.

This Special Issue also contains a review on the histidine methyltransferase SETD3. Witecka et al. [28] describe and discuss structural, biochemical, and functional aspects of SETD3 and highlight its role in actin polymerization and pathological conditions such as cancers.

We envisage that this Special Issue will be of interest to researchers of the protein methylation field and will promote further research on the writers of protein methylome and the functional outcomes of these chemical modifications.
